# Safety, tolerability and pharmacokinetics of emodepside, a potential novel treatment for onchocerciasis (river blindness), in healthy male subjects

**DOI:** 10.1111/bcp.14816

**Published:** 2021-03-31

**Authors:** Jean‐Yves Gillon, Jeremy Dennison, Frans van den Berg, Sophie Delhomme, Karen Dequatre Cheeseman, Claudia Peña Rossi, Nathalie Strub Wourgaft, Sabine Specht, Belén Pedrique, Frédéric Monnot, Susanne Skrabs, Maria‐Luisa Rodriguez, Heino Stass

**Affiliations:** ^1^ Drugs for Neglected Diseases initiative (DNDi) Geneva Switzerland; ^2^ Hammersmith Medicines Research Ltd London UK; ^3^ Invicro, Burlington Danes Building Imperial College London, Hammersmith Hospital London UK; ^4^ Present address: Bayer AG Wuppertal Germany

**Keywords:** emodepside, healthy subjects, onchocerciasis, pharmacokinetics, river blindness, safety

## Abstract

**Aims:**

Emodepside is an anthelmintic, originally developed for veterinary use. We investigated in healthy subjects the safety, and pharmacokinetics of a liquid service formulation (LSF) and immediate release (IR) tablet of emodepside in 2 randomised, parallel‐group, placebo‐controlled, Phase I studies.

**Methods:**

Seventy‐nine subjects in 10 cohorts in the single ascending dose study and 24 subjects in 3 ascending‐dose cohorts in the multiple ascending dose study were enrolled. Emodepside as LSF was administered orally as single 1–40‐mg doses and for 10 days as 5 or 10 mg once daily and 10‐mg twice daily doses, respectively. Pharmacokinetics and safety were assessed up to 21 and 30 days, respectively. In addition, IR tablets containing 5 or 20 mg emodepside were tested in the single ascending dose study.

**Results:**

Emodepside as LSF was rapidly absorbed under fasting conditions, with dose‐proportional increase in plasma concentrations at doses from 1 to 40 mg. Terminal half‐life was > 500 hours. In the fed state, emodepside was absorbed more slowly but overall plasma exposure was not significantly affected. Compared to the LSF, the rate and extent of absorption was significantly lower with the tablets.

**Conclusions:**

Overall, emodepside had acceptable safety and tolerability profiles, no major safety concerns, after single oral administration of 20 mg as LSF and after multiple oral administration over 10 days at 5 and 10 mg OD and at 10 mg twice daily. For further clinical trials, the development of a tablet formulation overcoming the limitations observed in the present study with the IR tablet formulation is considered.

**What is already known about the subject**

- Emodepside is an anthelmintic agent, currently registered for veterinary use in combination with other drug substances.
- Emodepside may have the potential to treat parasitic infections in humans, including onchocerciasis.

**What this study adds**

- No major safety concerns were identified in 103 healthy male subjects exposed to emodepside orally as a liquid service formulation up to 40‐mg single dose and 10 mg twice daily for 10 days.
- Emodepside applied as liquid service formulation had a favourable pharmacokinetics profile with roughly dose‐proportional C_max_ and AUC after up to 40‐mg dose.
- Tissue distribution was relatively rapid, which, with a long terminal half‐life, are expected to be beneficial for treatment of onchocerciasis.

## 1 INTRODUCTION

Onchocerciasis (river blindness) is a neglected tropical disease caused by *Onchocerca volvulus*, a parasitic nematode transmitted to humans through the bite of the blackfly.^1^ The larvae mature into reproductively competent adults within 1 year. Adult worms have a lifespan of 9–11 years and reside primarily in subcutaneous and deep‐tissue nodules where they produce offspring (microfilariae), which migrate to the skin awaiting uptake by another blackfly. The disease results from the death of the microfilariae, which prompts an inflammatory response, causing skin rash and lesions, including skin depigmentation, and unbearable itching. Microfilariae also migrate to the eye, causing local inflammation and other complications, including eye lesions, often leading to blindness.^2^ Onchocerciasis is endemic in 27 countries mainly in sub‐Saharan Africa, as well as in Yemen and Latin America.^1^

Onchocerciasis treatment and control currently rely on mass drug administration (MDA) of ivermectin (Mectizan, Merck & Co. Inc.), which targets the microfilarial stage of the parasite and temporarily sterilises, but does not kill, the adult worms. MDA programmes must therefore be repeated at regular intervals for many years, which represents a considerable economic and logistical burden in endemic countries.^3^ There is also mounting evidence of potential resistance to ivermectin.^4^ Another avermectin parasiticide, moxidectin, with prolonged efficacy compared to ivermectin was approved in 2018. Like ivermectin, however, it targets only microfilariae.^5^

Thus, there is an urgent need for new agents against onchocerciasis. Ideally, such agents should have activity against multiple life‐stages of the parasite, a good safety profile and a long‐lasting effect with a relatively simple dosing regimen. With ivermectin in place, it is not feasible to aim for replacement of current MDA drugs. However, a new macrofilaricidal drug would be an asset in focused MDA treatment or test‐and‐treat strategies for patients in endemic areas, where repeated ivermectin distribution is difficult or remains ineffective. In addition, the access to a macrofilaricidal drug for individual case management is an important goal for drug development in onchocerciasis. The generic Target Product Profile of such new agents is available on the website of Drugs for Neglected Diseases *initiative* (www.dndi.org/diseases/filaria-river-blindness/target-product-profile/).

Emodepside, a semi‐synthetic cyclo‐octadepsipeptide, is active across multiple nematode species. Like ivermectin and moxidectin,^6^, ^7^ emodepside was originally developed as an anthelmintic for veterinary use. It was first marketed as Profender (Bayer AG, Leverkusen, Germany) in 2005, in combination with praziquantel, and subsequently as Procox (Bayer AG, Leverkusen, Germany), in combination with toltrazuril.

Because of its unique, although not fully elucidated mechanism of action relative to other anthelmintics, emodepside is active at various stages in the nematode life‐cycle.^8^ Emodepside interacts with SLO‐1,^9^ a calcium activated potassium channel, which finally results in flaccid paralysis of the parasites (inhibition of locomotion, feeding, egg‐laying and slowed development). In gastrointestinal nematodes, it has been shown that emodepside also interacts with the g‐protein coupled receptors latrophilin LAT‐1,^10^ which is responsible for the paralytic effects on the worm pharynx.^11^ Preclinical pharmacology studies using in vitro and in vivo models of human filarial infections, including onchocerciasis, showed that emodepside was consistently active on parasites across several species and stages^12^ and is thus a potential candidate for human use. It is clear from these examples, that the effect on microfilariae is expected to be related to C_max_, whereas macrofilaricidal effects require a certain time over threshold for efficacy, as is the case for other tissue dwelling parasites. Additional nonclinical pharmacology information for emodepside efficacy against gastrointestinal nematodes is available in the literature.^12^, ^13^, ^14^, ^15^, ^16^ Also, emodepside meets criteria of the Target Product Profile for river blindness. Based on the evidence in animals, including favourable pharmacokinetics (PK) in various species and efficacy on filarial parasites,^15^, ^16^ evaluation of emodepside in human was considered in the perspective of developing it as a macro‐ and microfilaricidal treatment of onchocerciasis.

Here we report the basic PK including biopharmaceutical features and safety of emodepside in healthy male subjects after single (NCT02661178) and multiple (NCT03383614) oral doses as a liquid service formulation (LSF). As such a formulation would not be practical for use in the field in countries where river blindness is endemic, the safety and relative bioavailability of a standard immediate release tablet containing crystalline emodepside were also investigated. In addition, preliminary data on the effect of a standard Food and Drug Administration meal on the PK of emodepside are also described.

## 2 METHODS

### 2.1 General

The study designs are presented in Table 1. The 2 studies were approved by local research ethics committees in the UK and were conducted in compliance with the Declaration of Helsinki and the International Conference on Harmonisation E6 Guideline for Good Clinical Practice. A clinical trial authorisation was obtained from the Medicines and Healthcare products Regulatory Agency (UK) for each study. The studies were registered at clinicaltrials.gov and in EudraCT. Written informed consent was obtained from all subjects before undertaking any study‐related procedures. Quality assurance, data management and study monitoring were performed by contract research organisations (Hammersmith Medicines Research, London UK and Niche Science and Technology, Richmond, UK).

**TABLE 1:** Overview of the 2 Phase I studies on emodepside

	SAD study (first‐in‐human study)	MAD study
**Design features**	Two‐part, single‐centre, double‐blind, randomised, placebo‐controlled, parallel group, single ascending dose, comparative study	Single‐centre, double‐blind, randomised, placebo‐controlled, parallel‐group, multiple ascending dose study
**Study groups**	10 cohorts of 8 subjects each; 6 on emodepside, 2 on placebo	3 cohorts of 8 subjects each; 6 on emodepside, 2 on placebo
**Study population**	Healthy male subjects aged 18–55 y	Healthy male subjects aged 18–45 y
**Objectives**	Cohorts 1 to 8: Assess safety, tolerability and PK of single ascending oral doses Cohort 9: Assess food effect on bioavailability of LSF Cohort 10: Explore relationship between emodepside and AEs reported in part 1, in particular ophthalmological events	Assess safety, tolerability, PK and PD of multiple ascending oral doses of LSF over 10 days
**Doses studied**	Cohorts 1 to 8: LSF at 1 mg, 2.5 mg, 5 mg, 10 mg, 20 mg or 40 mg under fasting conditions; IR tablet: 5 mg or 20 mg under fasting conditions; Cohort 9: LSF at 10 mg under fed conditions; Cohort 10: LSF at 40 mg under fasting conditions	Cohort 1: 5 mg once daily for 10 days; cohort 2: 10 mg once daily for 10 days; cohort 3: 10 mg twice daily for 10 days (single intake on last day)
**Dose escalation**	Upon safety committee decision	Upon safety committee decision

AE: adverse event; IR: immediate release; LSF: liquid service formulation; MAD: multiple ascending dose; PD: pharmacodynamics; PK: pharmacokinetics; SAD: single ascending dose.

### 2.2 Investigational products

Emodepside and placebo were supplied as a LSF and a standard immediate‐release (IR) tablet. The LSF was a 0.1% (w/v) solution containing 1 mg emodepside/mL. Conventional IR tablets containing emodepside in crystalline form were supplied in 2 dosage strengths, 5 and 20 mg, for the single ascending dose (SAD) study.

The LSF, tablets and matching placebos were developed and manufactured by Bayer AG. Manufacturing, packaging, quality control and preparation of clinical supplies complied with Good Manufacturing Practice.

Randomisation, using a predetermined randomisation list and investigational product (IP) allocation, was performed by research personnel not involved in any other study‐related activity.

### 2.3 Subjects

At screening, subjects were deemed healthy based on medical history, physical examination, electrocardiography, vital signs and laboratory tests. Key exclusion criteria included presence or history of severe allergies, recent use of any prescription medicine, blood loss >400 mL or participation in another clinical study in the past 3 months.

To preclude any dietary effects on the PK of emodepside, subjects in the fasting cohorts fasted for 9 hours before receiving the IP. Subjects in the fed cohort fasted for 10 hours prior to dosing and received a standard high‐calorie, high‐fat breakfast 30 minutes prior to dosing.

### 2.4 PK analyses

Blood samples (5 mL) were collected by venepuncture or via a cannula into EDTA tubes and immediately placed on ice. Samples were centrifuged at 1500 *g* for 10 minutes at 4°C. The plasma was aliquoted into 2 polypropylene tubes, which were stored at −20°C. Blood samples for PK analysis were taken at the following time points:

• SAD: predose and 0.5, 1, 1.5, 2, 2.5, 3, 4, 5, 6, 8, 12, 24, 36, 48, 72, 96, 120, 144 and 168 hours, and 21 days postdose (cohort 1–9) or predose and 0.5, 1, 3, 6, 8, 12, 24, 36, 48, 72, 96, 120, 144 and 168 hours, and 10, 14, 18 and 21 days postdose (cohort 10).
• Multiple ascending dose (MAD), as below:
  • on Day 1: predose and at 0.25, 0.5, 1, 1.5, 2, 2.5, 3, 4, 6, 8, 12 and 15 hours postdose
  • on Days 2–9: before the morning dose
  • on Day 10: before and at 0.25, 0.5, 1, 1.5, 2, 2.5, 3, 4, 6, 8, 12 and 15 hours
  • on Days 11, 12, 13, 14, 15, 18, 21, 24 and 30.

Emodepside plasma concentrations were determined by a validated high performance liquid chromatography–tandem mass spectrometry assay, using a Alltima C18 5 μm 150 mm x 2.1 mm column and an Applied Biosystems API4000 Turbo Ion Spray detector using positive ion mode. Analyst version 1.3.2 or 1.6.3 software, obtained from AB Sciex UK Ltd, Warrington, UK was used for chromatogram data analysis and quantitative calculations. Deuterated emodepside‐D_16_ was used as the internal standard. The lower limit of quantitation for emodepside in plasma was 1 ng/mL. Values below this limit were not used to calculate the PK parameters, except values that were below the limit of quantification before C_max_, which were set to zero.

The highest observed plasma concentration (C_max_) was determined directly from concentration–time data, as was the time to reach maximum plasma concentration (t_max_). The area under the plasma concentration–time curve from time zero (predose) to the time of last quantifiable concentration (AUC_0‐last_) was calculated using the linear‐log trapezoidal rule. Elimination half‐life (t_1/2_) was calculated by the equation ln2/λ_z_.

### 2.5 Safety assessments

Safety was assessed by monitoring adverse events (AEs) throughout the studies that were coded using the standard Medical Dictionary for Regulatory Activities (MedDRA) dictionary (versions 19.0 [SAD part 1], 20.0 [SAD part 2] and 21.1 [MAD]). Other safety monitoring included 12‐lead electrocardiography recordings, measurement of vital signs (systolic and diastolic blood pressure and heart rate), physical and neurological (Hamilton depression rating scale^17^ and Beck depression inventory‐II fastscreen^18^ [SAD only], mental status examination, cranial nerves assessment and motor system examination, sensation to light touch, coordination/cerebellar function assessment, Romberg's test, gait test, daytime sleepiness assessment [SAD and MAD studies]) examinations, haematology, biochemistry and urinalysis. To minimise risks in the SAD and MAD studies, subjects were dosed sequentially using sentinels in each cohort (1 IP, 1 placebo) as per European Medicines Agency guidelines.^19^ If the previous dose was well tolerated, with no safety concerns, dose escalation was decided by the Safety Review Group after reviewing safety and PK data from all available cohorts. Moreover, treatment with the highest dose in the SAD study was repeated (cohort 10) for regular ophthalmological evaluations (assessments available as supplementary information) to better characterise visual AEs observed in the SAD study. Regular ophthalmological assessments were also performed in the MAD study.

### 2.6 Statistical analyses

Statistical analyses were performed using SAS version 9.3 (Cary, NC, USA). Demographic data and baseline characteristics were listed and summarised. Safety data did not undergo formal statistical analysis. PK parameters were derived from plasma concentration *vs*. time data using a noncompartmental analysis in Phoenix WinNonlin version 7 (Certara Inc., Princeton, NJ, USA). Plasma concentration *vs*. time data and PK parameters were listed, and summarised by treatment, using descriptive statistics. Mean concentrations were calculated only if at least ⅔ of the individual concentrations were above the lower limit of quantification. Individual subject and mean plasma concentrations were displayed graphically. Planned sampling times were used to summarise plasma‐concentration data; actual sampling times were used in the derivation of PK parameters.

### 2.7 Nomenclature of targets and ligands

Key protein targets and ligands in this article are hyperlinked to corresponding entries in http://www.guidetopharmacology.org, the common portal for data from the IUPHAR/BPS Guide to Pharmacology.

## 3 RESULTS

### 3.1 Subject disposition

Demographic characteristics were consistent across the 2 studies (Table 2). In the SAD study, 1 subject in the 1 mg LSF cohort was withdrawn from the study due to an AE. He received an incomplete dose of 0.1 mg emodepside in error. He was included in the safety assessment, but not the PK assessments. Only 5 of the planned 6 subjects were included in the 5‐mg IR tablet cohort. In the MAD study, all subjects received the IP as intended. The initial doses were selected based on the predicted human PK and therapeutic dose, derived from data obtained from in vitro and in vivo studies after administration of emodepside to rats and dogs and from in vitro data on plasma‐protein binding and blood–plasma partitioning.

**TABLE 2:** Demographic characteristics of subjects in the 2 Phase I studies on emodepside

Variable	Statistics	SAD study		MAD study
		Part 1 *n* = 63	Part 2 *n* = 16	*n* = 24
**Sex**	Male *n* (%)	63 (100)	16 (100)	24 (100)
**Age (y)**	Mean (SD)	32.4 (8.89)	34.4 (11.05)	31.3 (8.03)
	Median (range)	32 (19–54)	34.0 (21–52)	31.0 (19–43)
**Ethnicity**	White *n* (%)	60 (95.2)	15 (93.8)	24 (100)
	Hispanic or Latino *n* (%)	3 (4.8)	1 (6.3)	0
**Weight (kg)**	Mean (SD)	78.4 (9.9)	81.4 (11.6)	74.9 (10.8)
	Median (range)	76.6 (57.2–97.6)	81.5 (60–101.2)	74.05 (54.2–95.2)
**BMI**	Mean (SD)	24.4 (2.4)	25.2 (3.1)	22.9 (2.7)
	Median (range)	24.1 (19.0–29.7)	24.9 (20.5–29.9)	22.55 (18.1–27.8)
**Tobacco use**	*n* (%)	6 (9.5)	1 (6.3)	4 (16.7)
**Alcohol use**	*n* (%)	50 (79.4)	11 (68.7)	15 (62.5)

BMI: body mass index; MAD: multiple ascending dose; SAD: single ascending dose; SD: standard deviation.

### 3.2 PK parameters

#### 3.2.1 SAD and food effect study

**FIGURE 1:**
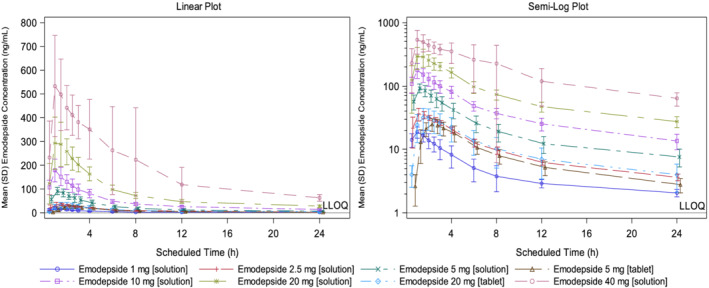
Geometric mean plasma emodepside concentration *vs*. time profiles after single oral administration of ascending doses from 1 to 40 mg as LSF or of 5 and 20 mg IRTs. LLOQ: lower limit of quantitation; LSF: liquid service formulation; IRT: immediate release tablet. Error bars represent geometric standard deviation

Mean plasma emodepside concentration–time profiles are shown in Figure 1 and PK parameters are presented in Table 3. Across all doses and for both formulations, after single administration, emodepside concentrations were rapidly quantifiable in the plasma, starting with the first timepoint at 0.5 hours postdose. Median t_max_ in subjects in the fasting state was shorter for the LSF than for the IR tablet. Exposure, based on C_max_ and AUC_0–24_ was dose‐proportional with the LSF up to the 40‐mg dose, but less than dose proportional with the IR tablet. The relative bioavailability of the tablet *vs*. the LSF was 35.0% for the 5‐mg dose and 11.7% for the 20‐mg dose (Table 4).

**TABLE 3:** Mean pharmacokinetic parameters for emodepside in the single ascending dose study

	1 mg LSF fasting	2.5 mg LSF fasting	5 mg LSF fasting	10 mg LSF fasting	20 mg LSF fasting	40 mg LSF fasting	5 mg IRT fasting	20 mg IRT fasting	10 mg LSF Fed
**AUC**_**last**_ (h.ng/mL)	182	845	1700	3070	7480	16 400	501	667	3390
	111	10.4	24.2	27.7	22.1	23.6	76.8	125	20.4
**AUC**_**0–24**_ (h.ng/mL)	100	250	522	996	1910	4110	183	223	673
	50.4	6.5	25.8	21.2	16.3	33.6	24.3	58.0	26.4
**AUC**_**0–24**_ **/D** ([h.ng/mL]/mg)	100	100	104	99.6	95.3	103	36.5	11.2	67.3
	50.4	6.5	25.8	21.2	16.3	33.6	24.3	58.0	26.4
**C**_**max**_ (ng/mL)	18.6	37.6	92.1	172	306	595	25.7	30.2	71.9
	20.8	15.5	16.2	32.3	28.7	27.9	23.9	62.5	29.6
**C**_**max**_ **/D** ([ng/mL]/mg)	18.6	15.0	18.4	17.2	15.3	14.9	5.15	1.51	7.19
	20.8	15.5	16.2	32.3	28.7	27.9	23.9	62.5	29.6
**t**_**max**_ (h)	1.00 (1.00–1.05)	1.00 (1.00–2.50)	1.00 (1.00–1.50)	1.00 (1.00–1.00)	1.50 (1.00–2.53)	1.05 (1.00–8.00)	2.00 (1.02–2.55)	2.00 (1.50–2.02)	2.50 (2.00–2.52)
**Terminal t**_**½**_ (h)	42.7	449	415	365	590	392	267	348	531
	531	74.0	117	286	68.1	31.7	392	171	99.3
**t**_**½ 0–24**_ ^a^ (h)	8.45	10.6	11.6	10.9	10.5	11. 1	10.8	11.3	11.1
	84.7	24.9	21.7	26.8	28.7	24.7	9.2	23.8	33.9

Note: all values are mean/CV%, except t_max_, which is median (range).

IRT: immediate release tablet; LSF: liquid service formulation.

^a^ Dominant half‐life, defined as half‐life calculated from the terminal slope of the log concentration–time (0–24 h) curve.

**TABLE 4:** Relative bioavailability of emodepside immediate release (IR) tablet compared to liquid service formulation (LSF) in the single ascending dose study

Dose	Geometric mean AUC_0–24_		IR tablet *vs*. LSF	
	IR tablet	LSF	F_rel_ (%)	90% CI
5 mg, fasting	182.5	521.9	34.97	26.56–46.03
20 mg, fasting	223.2	1905.8	11.71	7.73–17.75

AUC_0–24_: dose normalised area under the concentration–time curve from 0 to 24 hours; CI: confidence interval; F_rel_: relative bioavailability; IR: immediate release; LSF: liquid service formulation.

In the fed state, after a single 10‐mg dose of the LSF, geometric mean C_max_ and AUC_0–24_ were lower and median t_max_ was longer than after the same dose in the fasting state, indicating delayed absorption of emodepside; However, AUC_0‐last_ was not statistically different in fed relative to fasting conditions (Table 3).

Geometric mean elimination t_1/2_ at all dose levels and for both formulations was very long, while geometric mean t_1/2_ during the first 24 hours postdose was much shorter. Indeed, plasma emodepside concentrations were approximately 90% lower, based on geometric mean C_max_, in the first 24 hours postdose.

#### 3.2.2 MAD study

**FIGURE 2:**
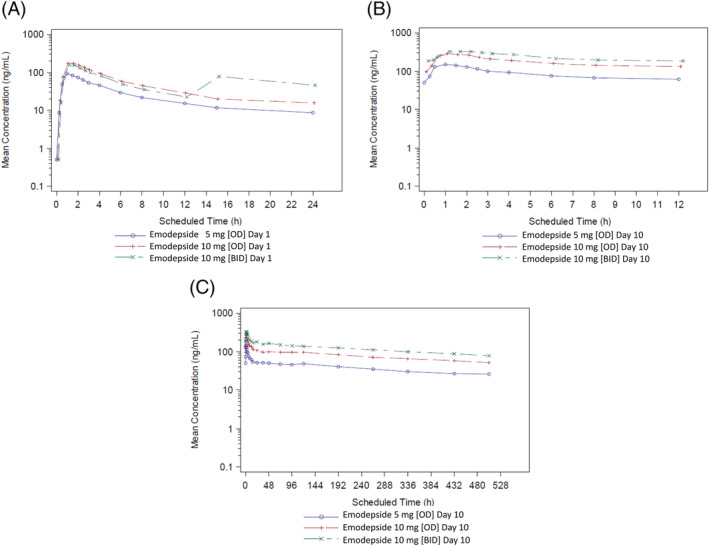
Geometric mean plasma emodepside concentration *vs*. time profiles after multiple oral administration of ascending doses of 5 mg once daily (OD), 10 mg OD or 10 mg twice daily (BID) as liquid service formulation. (A) Geometric mean plasma emodepside concentration *vs*. time profiles up to 24 hours after dosing on Day 1. (B) Geometric mean plasma emodepside concentration *vs*. time profiles up to 12 hours after dosing on Day 10. (C) Geometric mean plasma emodepside concentration *vs*. time profiles up to 528 hours after dosing on Day 10

Only LSF formulation was used in that study. Mean plasma emodepside concentration–time profiles are shown in Figure 2 and PK parameters are presented in Table 5. Rapid absorption of emodepside and the median t_max_ seen in the SAD study were confirmed across all dosing groups and regimens in the MAD study. Emodepside levels were still quantifiable in all subjects at the final sampling timepoint, 507 hours after the last morning dose, which was consistent with the findings in the SAD study.

**TABLE 5:** Mean pharmacokinetic parameters for emodepside in the multiple ascending dose study

	5 mg OD LSF fasting		10 mg OD LSF fasting		10 mg BID LSF fasting	
	Day 1	Day 10	Day 1	Day 10	Day 1	Day 10
**AUC**_**last**_ (h.ng/mL)	– (−)	19 359 (29.9)	– (−)	40 655 (43.5)	– (−)	59 554 (29.1)
**AUC**_**last**_ **/D** ([h.ng/mL]/mg)	– (−)	3872 (29.9)	– (−)	4065 (43.5)	– (−)	5955 (29.1)
**AUC**_**0–24**_ (h.ng/mL)	574 (19.7)	1689 (31.3)	1135 (32.7)	3487 (44.2)	1428 (26.5)	4897 (35.8)
**AUC**_**0–24**_ **/D** ([h.ng/mL]/mg)	115 (19.7)	338 (31.3)	113 (32.7)	349 (44.2)	71.4 (26.5)	490 (35.8)
**C**_**max**_ (ng/mL)	93.8 (17.8)	149 (17.9)	186 (21.3)	287 (39.7)	160 (20.4)	349 (27.1)
**C**_**max**_ **/D** ([ng/mL]/mg)	18.8 (17.8)	29.9 (17.9)	18.6 (21.3)	28.7 (39.7)	16.0 (20.4)	34.9 (27.1)
**C**_**trough**_ ^a^ (ng/mL)	– (−)	49.7 (36.8)	– (−)	97.1 (50.8)	– (−)	185 (39.5)
**t**_**max**_ (h)	1.00 (1.00–1.07)	1.00 (1.00–1.50)	1.25 (1.00–2.00)	1.25 (1.00–2.00)	1.00 (1.00–1.58)	1.50 (1.03–2.50)
**Terminal t**_**½**_ (h)	– (−)	419 (42.6)	– (−)	450 (30.6)	– (−)	508 (56.9)
**t**_**½ 0–24**_ ^b^ (h)	– (−)	26.9 (52.4)	– (−)	18.4 (30.0)	– (−)	33.2 (55.0)
**λ**_**z**_ (1/h)	– (−)	0.00166 (42.6)	– (−)	0.00154 (30.6)	– (−)	0.00137 (56.9)
**CL**_**ss**_ **/F** (L/h)	– (−)	2.96 (31.3)	– (−)	2.87 (44.2)	– (−)	3.56 (35.0)
**V**_**z**_ **/F** (L)	– (−)	1788 (74.2)	– (−)	1861 (68.5)	– (−)	2607 (102.1)
**MRT**_**last**_ (h)	7.28 (10.7)	– (−)	7.00 (11.0)	– (−)	10.8 (2.8)	– (−)

Note: all values are mean/CV%, except t_max_, which is median (range).

LSF: liquid service formulation; OD: once daily; BID: twice daily.

^a^ Before the final intake on Day 9.

^b^ Dominant half‐life, defined as half‐life calculated from the terminal slope of the log concentration–time (0–24 hr) curve.

C_max_ and AUC increased in a dose‐proportional manner after once daily (OD) dosing of 5 and 10 mg emodepside LSF. Exposure was higher after dosing with 10 mg twice daily (BID) than with 10 mg OD. Compared to predose concentrations on Days 2–9, C_trough_ levels in the 5 mg OD, 10 mg OD and 10 mg BID groups before the last dose on Day 10 indicated that steady state had still not been reached.

Elimination t_½_ was independent of dose with a geometric mean terminal t_½_ on Day 10 of 419 hours in the 5 mg OD group, 450 hours in the 10 mg OD group and 508 hours in the 10 mg BID group (Table 5). Plasma concentrations declined from C_max_ more rapidly during the 24 hours postdose than subsequently, again consistent with the findings in the SAD study.

The increase in plasma emodepside concentrations after the last dose on Day 10 was lower after 10 mg BID than after 10 mg OD, with C_max_/C_trough_ ratios of 1.9 and 3.0, respectively. Although the total daily dose in the 10 mg BID group was double that in the 10 mg OD group on Days 1–9, geometric mean C_max_ on Day 10 was only 1.2‐fold higher in the 10 mg BID group than in the 10 mg OD group.

### 3.3 Safety

Safety monitoring across all 2 studies did not identify any major concerns. Only 1 serious AE occurred in the MAD study, an abscess requiring hospitalisation for surgery, but was not considered treatment‐related. Mild to moderate non‐serious treatment‐related, treatment‐emergent AEs (TEAEs) were reported in the 2 studies. A TEAE was defined as an event that emerged during treatment and having been absent pretreatment, or that worsened relative to the pretreatment state.^20^

In the SAD, the onset of TEAEs involving visual disorders occurred at approximately t_max_, but with no clear evidence that they were directly related to plasma emodepside concentrations, since the duration ranged from 1 hour to 1 day. Drug‐related visual disorder TEAEs (photophobia, blurred vision, decreased visual acuity, altered perception of dimension, distorted colour perception, visual flashes) were reported after doses of 10 mg (2 subjects), 20 mg (1 subject) and 40 mg (5 subjects) LSF in the fasting state (Table 6) but were all mild and resolved spontaneously within 24 hours. Onset ranged from 20 minutes to 4 hours post‐dose, most often 1–2.5 hours postdose. The occurrence of drug‐related visual disorder TEAEs increased with emodepside dose.

**TABLE 6:** Treatment‐emergent adverse events reported with emodepside and placebo in the single ascending dose study, presented by system organ class

	Part 1											Part 2			
System organ class	Placebo LSF/IRT *n* = 16, *n* (%)	0.1 mg LSF *n =* 1, *n* (%)^a^	1 mg LSF *n =* 5, *n* (%)	2.5 mg LSF *n =* 6, *n* (%)	5 mg LSF *n =* 6, *n* (%)	5 mg IRT *n =* 5, *n* (%)	10 mg LSF *n =* 6, *n* (%)	20 mg LSF *n =* 6, *n* (%)	20 mg IRT *n =* 6, *n* (%)	40 mg LSF *n =* 6, *n* (%)	All subjects *n =* 63, *n* (%)	Placebo LSF/IRT *n =* 4, *n* (%)	10 mg LSF (fed) *n =* 6, *n* (%)	40 mg LSF (fasting) *n =* 6, *n* (%)	All subjects *n =* 16, *n* (%)
**Any TEAE**	6 (37.5)	1 (100)	3 (60.0)	0	3 (50.0)	3 (60.0)	5 (83.3)	3 (50.0)	2 (33.3)	5 (83.3)	31 (49.2)	0	3 (50.0)	5 (83.3)	8 (50.0)
Nervous system disorders	2 (12.5)	0	2 (40.0)	0	1 (16.7)	1 (20.0)	1 (16.7)	1 (16.7)	1 (16.7)	3 (50.0)	12 (19.0)	0	2 (33.3)	4 (66.7)	6 (37.5)
Eye disorders	1 (6.2)	0	0	0	0	1 (20.0)	2 (33.3)	1 (16.7)	0	5 (83.3)	10 (15.9)	0	0	5 (83.3)	5 (31.3)
Infections and infestations	0	1 (100)	0	0	1 (16.7)	0	1 (16.7)	1 (16.7)	0	1 (16.7)	5 (7.9)	0	0	0	0
Musculoskeletal and connective tissue disorders	0	0	1 (20.0)	0	1 (16.7)	1 (20.0)	0	0	1 (16.7)	1 (16.7)	5 (7.9)	0	0	1 (16.7)	1 (6.3)
Respiratory, thoracic and mediastinal disorders	1 (6.2)	0	0	0	0	0	1 (16.7)	1 (16.7)	1 (16.7)	0	4 (6.3)	0	1 (16.7)	1 (16.7)	2 (12.5)
Gastrointestinal disorders	2 (12.5)	0	0	0	1 (16.7)	0	0	0	1 (16.7)	0	4 (6.3)	0	0	3 (50.0)	3 (18.8)
General disorders and administration site disorders	0	0	0	0	2 (33.3)	0	0	0	0	0	2 (3.2)	0	0	3 (50.0)	3 (18.8)
Injury, poisoning and procedural complications	1 (6.2)	0	0	0	0	0	0	1 (16.7)	0	0	2 (3.2)	0	0	0	0
Psychiatric disorders	0	0	0	0	0	1 (20.0)	0	0	0	0	1 (1.6)	0	0	1 (16.7)	1 (6.3)

IRT: immediate‐release tablet; LSF: liquid service formulation.

Subjects with ≥1 adverse event are counted only once per system organ class and preferred term.

^a^ One subject received 0.1 mg emodepside LSF, which was recorded as a protocol deviation.

Cases involving transient, mild visual disturbances (visual impairment, vivid vision), considered drug‐related, also occurred in the MAD study in all treatment groups, frequently associated with mild euphoria (Table 7). One subject treated with the lower dose also reported blurred vision of moderate intensity. Unlike in the SAD study, there was no clear relationship between the frequency of visual AEs and the dose of emodepside up to 10 mg BID; however, the duration of visual AEs was longer after 10 mg emodepside BID, with intermittent symptoms recurring up to 21 days. Based on these observations, 10 mg emodepside BID was considered to be the maximum tolerated dose as the LSF in the MAD study.

**TABLE 7:** Treatment‐emergent adverse events (TEAEs) reported with emodepside and placebo in the multiple ascending dose study, presented by system organ class

		Emodepside			
System organ class	Placebo *n =* 6, *n (%)*	LSF 5 mg OD *n =* 6, *n (*%)^a^	LSF 10 mg OD *n =* 6, *n (*%)	LSF 10 mg BID *n =* 6, *n (*%)	All subjects *n =* 24, *n (*%)
**Any TEAE**	4 (66.7)	5 (83.3)	6 (100.0)	6 (100)	21 (87.5)
Infections and infestations	0	4 (66.7)	4 (66.7)	1 (16.7)	9 (37.5)
Eye disorders	1 (16.7)	3 (50.0)	1 (16.7)	3 (50.0)	8 (33.3)
Musculoskeletal and connective tissue disorders	0	2 (33.3)	2 (33.3)	1 (16.7)	5 (20.8)
Nervous system disorders	2 (33.3)	2 (33.3)	0	0	4 (16.7)
Gastrointestinal disorders	2 (33.3)	2 (33.3)	0	0	4 (16.7)
General disorders and administration site disorders	1 (16.7)	1 (16.7)	1 (16.7)	1 (16.7)	4 (16.7)
Psychiatric disorders	0	2 (33.3)	1 (16.7)	0	3 (12.5)
Skin and subcutaneous tissue disorders	0	1 (16.7)	1 (16.7)	0	2 (8.3)
Investigations	0	0	0	1 (16.7)	1 (4.2)
Immune system disorders	0	0	0	1 (16.7)	1 (4.2)
Injury, poisoning and procedural complications	0	0	0	1 (16.7)	1 (4.2)
Metabolism and nutrition disorders	0	0	1 (16.7)	0	1 (4.2)
Respiratory, thoracic and mediastinal disorders	0	1 (16.7)	0	0	1 (4.2)
Surgical and medicinal procedures	0	0	1 (16.7)	0	1 (4.2)

LSF: liquid service formulation; OD: once daily; BID: twice daily.

Subjects with ≥1 adverse event are counted only once per system organ class and preferred term.

## 4 DISCUSSION

De novo discovery and development of new therapeutics is an extremely costly and time‐consuming process that is rarely conducted for neglected tropical diseases, including onchocerciasis, where drug discovery is notoriously under‐funded. Since emodepside is a registered product in animal health, the present studies confirm the usefulness of drug repurposing as a strategy for identifying and developing new therapeutic agents. The safety and efficacy of emodepside in animals are well established after nearly 15 years of use in the veterinary setting. As expected, the early clinical development results in healthy male subjects from the 2 Phase I studies reported here show promising safety profiles and led to significant exposure.

Emodepside was found to be rapidly absorbed under fasting conditions in healthy subjects after single or multiple oral doses. Dose‐proportional increases in plasma emodepside concentrations were observed with increasing doses from 1 to 40 mg after single oral administration of the LSF. For logistical reasons, the liquid formulation would not be practical for use in the field in Phase II and III studies or when rolling out a macrofilaricidal drug in countries where onchocerciasis is endemic and therefore also a standard tablet formulation was tested. Relative to the LSF, standard tablets containing 5 mg crystalline emodepside had a low bioavailability that was further reduced with the 20 mg strength, suggesting absorption limitations. The underlying cause is likely to be a reduced solubility of crystalline emodepside. Therefore, this formulation will not be appropriate for further clinical studies aiming at determining dosing strategies, especially when higher doses are needed to reach the desired plasma levels for efficacy. Formulation development using amorphous emodepside is expected to provide tablets with significantly improved biopharmaceutical properties and higher bioavailability compared to the conventional IR tablets assessed in healthy subjects.

In the 2 studies, plasma concentration–time profiles for emodepside showed a distinct biphasic pattern in the descending part of the curve, which is postulated to reflect initial relatively rapid tissue distribution of the drug followed by very slow terminal elimination. The half‐life of emodepside during the first 24 hours after dosing, i.e. during the distribution phase mainly was relatively short at around 11 hours and was followed by a very long terminal elimination half‐life, estimated at >500 hours. This is in line with nonclinical PK studies in rats and dogs, in which emodepside volume of distribution was estimated to be high. This finding in humans is of particular importance since rapid distribution of emodepside in the tissues suggests that exposure of both macro‐ and microfilariae of *O. volvulus* parasites may be high, given that microfilariae reside mainly in the skin and adult worms in subcutaneous tissue. This hypothesis is backed up by preclinical findings with radio‐labelled emodepside, showing in rats that radioactivity levels were higher in most tissues than in the blood, the highest concentrations being detected in the fat at all time points. In addition, the long terminal half‐life, which significantly contributes to the total drug exposure is expected to be advantageous in maintaining patient exposure to pharmacodynamically active drug levels and may offer, for the dosing strategy, the possibility to use in onchocerciasis patients a loading dose to reach a significant time over threshold for efficacy against these tissue parasites. Such a long half‐life is not expected to raise any safety issues, based on the available toxicological data and the safety profile of emodepside across the 2 Phase I studies in healthy subjects. Owing to the long terminal elimination half‐life, steady state was not reached after repeated dosing with emodepside for 10 days in the MAD study. In that study, the increase in plasma emodepside concentration after the final dose of the LSF on Day 10 was lower after 10 mg BID than after 10 mg OD, with C_max_/C_trough_ ratios of 1.9 and 3.0, respectively. This suggested that twice‐daily dosing might be beneficial in achieving a favourable C_max_/C_trough_ ratio to optimize drug exposure for clinical use. Geometric mean C_max_ on Day 10 in the 10 mg BID dosing group was only 1.2‐fold higher in the 10 mg BID dosing group compared to the 10 mg OD group, suggesting more limited safety risk with respect to potential concentration‐related AEs, thereby improving tolerability.

After oral administration of 10 mg emodepside as LSF in fed condition, absorption was delayed with a longer median t_max_ compared to that after the same dose in fasting condition (2.5 vs 1.0 h), however food did not significantly alter total exposure as AUC_0‐tlast_ were 3390 and 3070 h.ng/mL, respectively.

No important safety risks, either potential or identified, have been identified with emodepside to date. Safety data accrued in the SAD and MAD studies indicate that potential effects on the central nervous system and vision will require close monitoring in future studies.

Thus, these Phase I studies provided invaluable information on the safety, PK profile and relative bioavailability of emodepside in healthy humans. The LSF is not suitable for easy and accurate clinical use under the conditions expected for patients treated for onchocerciasis, and further studies are on‐going to select a tablet formulation compatible with the biopharmaceutical properties of emodepside.

## COMPETING INTERESTS

The authors declare that they have no conflicts of interest.

## CONTRIBUTORS

Research design: J.Y.G., C.P.R., N.S.W.

Research performance: J.D., F.V.D.B., S.D., K.D.C., J.Y.G, F.M.

Data analysis: all authors.

Manuscript writing: J.Y.G., S.Sp.

Manuscript revision: all authors.

## Supporting information


**Data S1.** Supporting information

## Data Availability

The data that support the findings of this study are available on request from the corresponding author. The data are not publicly available due to privacy or ethical restrictions.
