# Supplementary material for: Safety, tolerability and pharmacokinetics of emodepside, a potential novel treatment for onchocerciasis (river blindness), in healthy male subjects
Source: Br J Clin Pharmacol. 2021 Mar 31;87(10):3949–60. doi: 10.1111/bcp.14816 (PMC8518114; doi:10.1111/bcp.14816)
Supplement: Supplementary file 1 — Data S1. Supporting information [file BCP-87-3949-s001.docx]

**Supplementary information**

Ophthalmological assessments performed in the single ascending dose study, Part 2, Cohort 10 and in the multiple ascending dose study:

• Ocular symptoms (abnormal coloration; sparkles of light in the vision; light sensitivity; disturbance of perception of straight lines; eye pain; double vision; and any other ocular symptoms)

• Past ocular history (use of contact lenses; any ongoing topical ocular therapy; history of ocular disease requiring ongoing treatment or surgery; and any previous ocular problems)

• Auto-refraction (using an autorefractor in an examination room with subdued lighting. The average of 3 readings was recorded for each eye)

• Best corrected distance visual acuity (assessed in each eye using an appropriately illuminated Snellen /LogMAR chart at an appropriate height in a room with moderate lighting)

• Colour vision assessment (using the Ishihara test (first 13 plates) in a room lit by daylight or by electric light adjusted as far as possible to resemble natural daylight)

• Amsler grid assessment (blurring or grey areas; distortion or wavy lines; holes or spots in any areas of the grid)

• Ocular alignment and ocular motility (esotropia; exotropia; hypertropia; speed, smoothness, range and symmetry of movements; unsteadiness of fixation; nystagmus; diplopia in each direction; saccades)

• Confrontation visual field assessment

• Slit lamp examination (anterior segment; test was done undilated then with mydriasis at screening and undilated only on Day 1)

- Post mydriatic ocular media (at Screening visit 2 only) and retinal examination with slit lamp and lens

Additional ophthalmological assessments performed in multiple ascending dose study only:

- Measurement of intraocular pressure
- Optical coherence tomography test
